# Case Report: Auricular vagus nerve stimulation possibly alleviates COVID-19 disease on a high-risk patient

**DOI:** 10.3389/fphys.2022.1000194

**Published:** 2023-01-12

**Authors:** József Constantin Széles, Felix Lucny, Alexander Tyercha, Eugenijus Kaniusas, Christoph Neumayer

**Affiliations:** ^1^ Center for Wound Surgery, Health Service Center of Vienna Privat Clinics, Vienna, Austria; ^2^ Department of General Surgery, Division of Vascular Surgery, Medical University of Vienna, Vienna, Austria; ^3^ Faculty of Electrical Engineering and Information Technology, Institute of Biomedical Electronics, Vienna University of Technology (TU Wien), Vienna, Austria

**Keywords:** COVID-19, SARS-CoV-2, auricular vagus nerve stimulation, high-risk patient, case report, aVNS

## Abstract

**Introduction:** SARS-CoV-2 is a highly contagious virus that was identified as the cause of COVID-19 disease in early 2020. The infection is clinically similar to interstitial pneumonia and acute respiratory distress syndrome (ARDS) and often shows cardiovascular damage. Patients with cardiovascular risk factors are more prone to COVID-19 disease and their sequelae. Due to the anti-inflammatory effect and the improvement in pulmonary function, auricular vagus nerve stimulation (aVNS) therapy might alleviate a COVID-19 infection.

**Patient and Methods:** A high-risk patient with cardiovascular diseases and Implantable Cardioverter Defibrillator (ICD), type 2 diabetes and peripheral arterial disease IV, according to Rutherford`s classification, became infected with COVID-19. The patient underwent wound surgery because of an infected necrosis with a methicillin-resistant *Staphylococcus aureus* (MRSA) of his small toe and was already on aVNS therapy to relieve his leg pain and improve microcirculation. AVNS was performed with the AuriStim device (Multisana GmbH, Austria), which stimulates vagally innervated regions of the auricle by administering electrical stimulation *via* percutaneous electrodes for 6 weeks.

**Results:** The multimorbid high-risk patient, who was expected to go through a severe course of the COVID-19 disease, showed hardly any symptoms during ongoing aVNS therapy, while other family members, being much younger and healthy suffered from a more serious course with headache, pneumonia and general weakness.

**Conclusion:** The auricular vagus nerve stimulation is a clinically tested and safe procedure and might represent an alternative and effective way of treating COVID-19 disease. Nevertheless, due to several limitations of this case report, randomized controlled studies are needed to evaluate the efficacy of aVNS therapy on COVID-19 disease.

## 1 Introduction

SARS-CoV-2 (severe acute respiratory syndrome coronavirus type 2) is a highly contagious virus that was identified as the cause of COVID-19 disease in early 2020 (Kaniusas et al., 2020). To enter the host cell, the virus uses the enzyme Angiotensin converting enzyme-2 (ACE-2) as a receptor. Clinically, the infection is mostly pulmonary, similar to interstitial pneumonia and acute respiratory distress syndrome and often leads to cardiovascular damage (Luo et al., 2021). In January 2021, there were approximately 94.7 million active cases and 2.2 million deaths (Organization, 2022).

The COVID-19 infection showed an increased risk of serious consequences including death in some concomitant diseases. An advanced patient age and cardiovascular diseases are responsible for a strong increase in risk. A higher mortality rate can be seen from the age of 65 and a significantly higher mortality rate from the age of 85. Other risk factors for a severe course can be found in Table 1 (Dayaramani et al., 2021). A recent study showed, that patients with cardiovascular disease often have a more severe course of COVID-19 disease and a higher mortality rate (Luo et al., 2021). The reason for this is the additional damage to the cardiovascular system caused by various mechanisms of infection.

**TABLE 1:** Risk factors for a severe course of COVID-19. PAD: Peripheral artery disease; MRSA: Methicillin resistant *Staphylococcus aureus* (Kaniusas et al., 2020; Dayaramani et al., 2021; Hu et al., 2021; Luo et al., 2021).

Diseases leading to a higher risk factor for a severe course of COVID-19
Hypertension
Impaired heart function (i.e., impaired left ventricular function due to a aortic valve insufficiency, myocardial infection)
High Patient Age
Circulatory disorders (i.e., PAD)
Simultaneous infection by other bacteria or viruses (i.e., MRSA)
Diabetes mellitus
Lung disease
Overweight/Obesity
Liver disease
Kidney disease
No vaccination

The neuromodulation through the auricular vagus nerve stimulation (aVNS) has gained increasing attention in various clinical pictures in recent years. The afferent auricular vagus nerve can be stimulated in the area of the concha using percutaneous stimulation needles resulting in activation of the cortex (Badran et al., 2018). No surgical intervention is necessary for this (Kaniusas et al., 2020) while aVNS is a clinically safe and tested procedure for the therapy of pain (Kaniusas et al., 2019a; Kaniusas et al., 2019b).

Several studies reviewed positive aVNS effects in patients with cardiovascular diseases, neurological disorders as well as inflammatory diseases (Kaniusas et al., 2019a; Kaniusas et al., 2019b; Kaniusas et al., 2020). The mechanisms behind the aVNS therapy are the anti-inflammatory and the vasodilative effects of the vagus nerve. Therefore, aVNS may represent a promising alternative to pharmacological therapies for various diseases including COVID-19, as hypothesized by our group (Kaniusas et al., 2020).

“AuriStim is a single-use, miniaturized, and battery-powered electrical stimulator. The stimulator delivers monophasic varying polarity pulses (pulse width 1 ms) with a fixed amplitude (3.8 V) every second (stimulation frequency is 1 Hz), and a duty cycle (3 h ON/3 h OFF). The resistance of the needle and needle to tissue interface is about 4–7kOhm so that the resulting peak current amount to about .5–.9 mA, residing at or below the suggested limits of about 1.5 mA” (Seitz et al., 2022).

## 2 Case report

### 2.1 Ethical issues

The case study is conducted in full compliance with the principles of the “Declaration of Helsinki” (as amended at the 64th WMA General Assembly, Fortaleza, Brazil, 2013) and follows Austrian laws and regulations as well as ICH GCP Guidelines. The AuriStim device (Multisana^©^) is a licensed product with very few adverse effects. The patient was fully advised of the risks of treatment. He gave his informed consent for treatment and his approval for publishing his case. In addition, the patient was informed about possible interactions of aVNS and ICD and signed a written consent for his treatment. Several experiments on the interaction of aVNS and ICD with electrocardiogram (ECG) motoring were made in other patients, which showed no interactions.

### 2.2 Patient

This study presents a male patient at the age of 81, who is being treated at the Wound Surgery Center due to multiple wounds and a chronic wound healing disorder. He had peripheral arterial disease (PAD) (stage IV according to Fontaine’s classification) with necrosis of his small toe. According to Table 1, the patient presented several risk factors. His secondary diseases, according to ICD-10 codes, are:

• E.11.5 Type 2 Diabetes mellitus with peripheral vascular complications
• E.11 Diabetic foot syndrome
• I70.25 PAD IV with necrosis in Digitus pedis V dextra
• I21.9 St. p. Myocardial infarction
• Z95.0 Presence of an Implantable Cardioverter Defibrillator (ICD)
• I10 Hypertension
• E78.0 Hypercholesterolemia

The family history was negative with regard to heart-/lung diseases as well as cancer. Both parents reached an age over 90 years.

### 2.3 Diagnostic assessment

During ongoing wound therapy and a chronic infection with MRSA the patient got infected with SARS-CoV-2 on 12 January 2021. He was tested positive with a PCR test for the mutation B.1.617.3 (known as Delta mutation). He was sent into isolation and tested negative after 14 days. Throughout the course of SARS-CoV-2 infection, the patient continued treatment with aVNS therapy once a week, keeping a regular correspondence with the doctor. Although a severe course was expected in this high-risk patient with diabetes, PAD, St. p. myocardial infarction and MRSA infection, he did not present any COVID-19 related symptoms. Infection parameters increased only moderately after 2 weeks (Table 2).

**TABLE 2:** History of the following blood values of the high-risk patient: C-reactive protein (CRP) and leucocytes. In red highlighted is the CRP increase during COVID-19 infection.

	19.12.2020	24.01.2021	13.02.2021	Unit	Reference
Leukocytes	7.1	6.8	8.2	G/l	4.0–10.0
CRP	0.1	0.8	0.9	mg/dl	<0.5

The patient took his prescribed pharmacological treatment with Acetylsalicylic acid 100 mg and Phenprocoumon 3 mg because of his cardiovascular diseases and PAD. This medication shows in a study from 2020 a lower incidence of pulmonary embolism (Haque et al., 2020). Due to the anticoagulation and platelet aggregation inhibitor medication, the patient did not suffer from any thrombotic events, as described in different studies during COVID-19 infection (Ali and Spinler, 2021; Gomez-Mesa et al., 2021).

Two other family members at the age of 34 and 48, which were vaccinated against COVID-19 in the first week of December 2020 with the first injection of Biontech/Pfizer. They did not present any secondary diseases and were also tested positive with a PCR-test for the same mutation of COVID-19 as our high-risk patient in the meantime. However, they suffered a more severe course, with heavy headaches, flu like symptoms, loss of taste and smell and arthralgia. Furthermore, significantly fewer antibodies were formed in these two family members compared to the high-risk patient, 2 months after the course of infection (Table 3).

**TABLE 3:** Comparison of COVID-19 antibodies between the high-risk patient and the family members.

	03.2021	05.2021	07.2021
Patient	1053.0 BAU/ml	—	417.0 BAU/ml
Family member 1	137.0 BAU/ml	20.0 BAU/ml	—
Family member 2	22.3 BAU/ml	—	—

BAU, binding antibody units.

## 3 Discussion

This case report illustrates a multi-morbid patient with a chronic wound infection (MRSA) in the right foot due to PAD and diabetes mellitus, who suffered from COVID-19 disease during wound surgical treatment. In addition to wound surgery, treatment included an aVNS therapy. Even though the patient presented multiple risk factors including cardiovascular disease, chronic wound infection, and a high age and was not vaccinated against COVID-19, he did not suffer from a severe course of the disease, while other vaccinated family members without secondary disease showed multiple and severe COVID-19 related symptoms.

A potential explanation for the mild course of COVID-19 in this high-risk patient is the activation of the parasympathetic system through ongoing aVNS therapy. As shown in several studies, this causes a stimulation of the anti-inflammatory pathway, avoiding cytokine storms, improving respiration, and causing a vasodilatation (Kaniusas et al., 2019a; Kaniusas et al., 2019b; Kaniusas et al., 2020). In recent years multiple studies had shown that aVNS is able to modulate inflammation *via* the cholinergic anti-inflammatory pathway in bacterial infections (Kaniusas et al., 2019a; Kaniusas et al., 2019b; Kaniusas et al., 2020). In our case study, aVNS seemed to manage the course of the COVID-19 disease in a high-risk patient. From a medical point of view, no further therapeutic measures were necessary.

A study on two case reports, published in 2020 discussed the symptomatic relief of respiratory symptoms during COVID-19 infection, like dyspnea and chest tightness as well as a reduction of cough suppressant medication (Staats et al., 2020). However, they used a device that stimulates the cervical branch of the vagus nerve. An explanation for a relief of respiratory symptoms through VNS is, that it inhibits through the parasympathetic stimulation a smooth muscle cell relaxation in the bronchi and bronchioles, causing a bronchodilation (Hoffmann et al., 2009).

Seitz et al. showed in 2022 that aVNS reduced the cytokine storm in COVID-19 patients as well as pro-inflammatory proteins, while anti-inflammatory cytokines increased during therapy (Seitz et al., 2022). Due to the good tolerance and the low risk of side effects, non-invasive auricular vagus nerve stimulation presents a good option for additional treatment in COVID-19 patients.

### 3.1 Limitation

This is a single case report, which limits the significance of this thesis. Furthermore, blood samples were limited to a basic hemogram with a few infection parameters. This could be extended to analyze different cytokines or immune cells in the blood, showing the systemic effects of aVNS therapy in COVID-19 patients. Another limitation of this case report is that it outlines only the clinical situation of the patient without a pathophysiological explanation for the outcome.

Furthermore, due to the multiple co-morbidities it is difficult to deduce a correlation between COVID-19 and aVNS therapy. Additionally, there is no control group, so it is not possible to accredit the aVNS therapy fully to the absence of severe symptoms during COVID-19 infection.

## 4 Conclusion

A multimorbid high risk patient suffered from COVID-19 during aVNS therapy. Because of a chronic wound infection due to PAD IV and diabetes the patient received aVNS. During ongoing aVNS therapy the patient did not present any COVID-19 related symptoms, although he was highly multimorbid and non-vaccinated. This observation encourages the hypothesis that aVNS might be a safe and effective way for the treatment of the COVID-19 infection. Nevertheless, due to the multiple co-morbidities of the patient as well as the absence of a control group, aVNS therapy cannot be fully correlated to the absence of the severe symptoms of the COVID-19 infection. This needs to be evaluated in cohort studies with a higher number of patients.

## Data Availability

The original contributions presented in the study are included in the article/Supplementary Material, further inquiries can be directed to the corresponding author.

## References

[B1] Ali M. A. M. Spinler S. A. (2021). COVID-19 and thrombosis: From bench to bedside. Trends Cardiovasc Med 31 (3), 143–160. 10.1016/j.tcm.2020.12.004 33338635 PMC7836332

[B2] Badran B. W. Dowdle L. T. Mithoefer O. J. LaBate N. T. Coatsworth J. Brown J. C., (2018). Neurophysiologic effects of transcutaneous auricular vagus nerve stimulation (taVNS) via electrical stimulation of the tragus: A concurrent taVNS/fMRI study and review. Brain Stimul 11 (3), 492–500. 10.1016/j.brs.2017.12.009 29361441 PMC6487660

[B3] Dayaramani C. De Leon J. Reiss A. B. (2021). Cardiovascular disease complicating COVID-19 in the elderly. Med. Kaunas 57 (8), 833. 10.3390/medicina57080833 PMC8399122 34441038

[B4] Gomez-Mesa J. E. Galindo-Coral S. Montes M. C. Munoz Martin A. J. (2021). Thrombosis and coagulopathy in COVID-19. Curr. Probl. Cardiol 46 (3), 100742. 10.1016/j.cpcardiol.2020.100742 33243440 PMC7605852

[B5] Haque S. Jawed A. Akhter N. Dar S. A. Khan F. Mandal R. K., (2020). Acetylsalicylic acid (aspirin): A potent medicine for preventing COVID-19 deaths caused by thrombosis and pulmonary embolism. Eur. Rev. Med. Pharmacol. Sci 24 (18), 9244–9245. 10.26355/eurrev_202009_23005 33015764

[B6] Hoffmann T. J. Mendez S. Staats P. Emala C. W. Guo P. (2009). Inhibition of histamine-induced bronchoconstriction in Guinea pig and Swine by pulsed electrical vagus nerve stimulation. Neuromodulation 12 (4), 261–269. 10.1111/j.1525-1403.2009.00234.x 22151415

[B7] Hu K. Lin L. Liang Y. Shao X. Hu Z. Luo H., (2021). COVID-19: Risk factors for severe cases of the Delta variant. Aging (Albany NY) 13 (20), 23459–23470. 10.18632/aging.203655 34710058 PMC8580340

[B8] Kaniusas E. Kampusch S. Tittgemeyer M. Panetsos F. Gines R. F. Papa M., (2019). Current directions in the auricular vagus nerve stimulation I - a physiological perspective. Front. Neurosci 13, 854. 10.3389/fnins.2019.00854 31447643 PMC6697069

[B9] Kaniusas E. Kampusch S. Tittgemeyer M. Panetsos F. Gines R. F. Papa M., (2019). Current directions in the auricular vagus nerve stimulation II - an engineering perspective. Front. Neurosci 13, 772. 10.3389/fnins.2019.00772 31396044 PMC6667675

[B10] Kaniusas E. Szeles J. C. Kampusch S. Alfageme-Lopez N. Yucuma-Conde D. Li X., (2020). Non-invasive auricular vagus nerve stimulation as a potential treatment for covid19-originated acute respiratory distress syndrome. Front. Physiol 11, 890. 10.3389/fphys.2020.00890 32848845 PMC7399203

[B11] Luo J. Zhu X. Jian J. Chen X. Yin K. (2021). Cardiovascular disease in patients with COVID-19: Evidence from cardiovascular pathology to treatment. Acta Biochim. Biophys. Sin. (Shanghai) 53 (3), 273–282. 10.1093/abbs/gmaa176 33428706 PMC7929476

[B12] Organization W. H. (2022). WHO coronavirus (COVID-19) dashboard. Geneva, Switzerland: World Health Organization (WHO).

[B13] Seitz T. Szeles J. C. Kitzberger R. Holbik J. Grieb A. Wolf H., (2022). Percutaneous auricular vagus nerve stimulation reduces inflammation in critical covid-19 patients. Front. Physiology 13, 897257. 10.3389/fphys.2022.897257 PMC9289290 35860660

[B14] Staats P. Giannakopoulos G. Blake J. Liebler E. Levy R. M. (2020). The use of non-invasive vagus nerve stimulation to treat respiratory symptoms associated with COVID-19: A theoretical hypothesis and early clinical experience. Neuromodulation 23 (6), 784–788. 10.1111/ner.13172 32342609 PMC7267613

