# Supplementary material for: Case Report: Auricular vagus nerve stimulation possibly alleviates COVID-19 disease on a high-risk patient
Source: Front Physiol. 2023 Jan 12;13:1000194. doi: 10.3389/fphys.2022.1000194 (PMC9877214; doi:10.3389/fphys.2022.1000194)
Supplement: Supplementary file 1 [file Table1.pdf]

Auricular vagus nerve stimulation possibly alleviates COVID-19 disease. A Case Report on a high-risk patient

Supplementary Material

1 Supplementary Tables

**Diseases leading to a higher risk factor for a severe course of COVID-19**
Hypertension
Impaired heart function (i.e. impaired left ventricular function due to a aortic valve insufficiency, myocardial infection)
High Patient Age
Circulatory disorders (i.e. PAD)
Simultaneous infection by other bacteria or viruses (i.e. MRSA)
Diabetes mellitus
Lung disease
Overweight/Obesity
Liver disease
Kidney disease
No vaccination

Table 1: Risk factors for a severe course of COVID-19. PAD: Peripheral artery disease; MRSA: Methicillin resistant Staphylococcus aureus (1, 2, 4, 8)

	19.12.2020	24.01.2021	13.02.2021	Unit	Reference
**Leukocytes**	7,1	6,8	8,2	G/l	4,0 - 10,0
**CRP**	0,1	0,8	0,9	mg/dl	< 0,5

Table 2: History of the following blood values of the high-risk patient: C-reactive protein (CRP) and leucocytes. In red highlighted is the CRP increase during COVID-19 infection.

	03.2021	05.2021	07.2021
**Patient**	1053,0 BAU/ml		417 BAU/ml
**Family member 1**	137 BAU/ml	20 BAU/ml	
**Family member 2**	22.3 BAU/ml		

Table 3: Comparison of COVID-19 antibodies between the high-risk patient and the family members. (BAU = binding antibody units)
